# Design and synthesis of novel oridonin analogues as potent anticancer agents

**DOI:** 10.1080/14756366.2017.1419219

**Published:** 2018-01-05

**Authors:** Qing-Kun Shen, Zheng-Ai Chen, Hong-Jian Zhang, Jia-Li Li, Chuan-Feng Liu, Guo-Hua Gong, Zhe-Shan Quan

**Affiliations:** a Key Laboratory of Natural Resources and Functional Molecules of the Changbai Mountain, Affiliated Ministry of Education, College of Pharmacy, Yanbian University, Yanji, China;; b Department of Pharmacology, Medical School of Yanbian University, Yanji, China;; c Medicinal Chemistry and Pharmacology Institute, Inner Mongolia University for the Nationalities, Tongliao, China;; d Inner Mongolia Autonomous Region Key Laboratory of Mongolian Medicine Pharmacology for Cardio-Cerebral Vascular System, Tongliao, China

**Keywords:** Oridonin, synthesis, anticancer, apoptosis

## Abstract

To identify anticancer agents with higher potency and lower toxicity, a series of oridonin derivatives with substituted benzene moieties at the C17 position were designed, synthesised, and evaluated for their antiproliferative properties. Most of the derivatives exhibited antiproliferative effects against AGS, MGC803, Bel7402, HCT116, A549, and HeLa cells. Compound **2p** (IC_50 _= 1.05 µM) exhibited the most potent antiproliferative activity against HCT116 cells; it was more potent than oridonin (IC_50_ = 6.84 µM) and 5-fluorouracil (5-FU) (IC_50 _= 24.80 µM). The IC_50_ value of **2p** in L02 cells was 6.5-fold higher than that in HCT116 cells. Overall, it exhibited better selective antiproliferative activity and specificity than oridonin and 5-FU. Furthermore, compound **2p** arrested HCT116 cells at the G2 phase of the cell cycle and increased the percentage of apoptotic cells to a greater extent than oridonin.

## Introduction

Over the last several decades, cancer has been one of the leading causes of death in the world^1^. Cytotoxic agents are the mainstay of anticancer therapy. However, due to their inability to differentiate between normal and cancerous cells, they cause severe adverse effects, leading to poor patient compliance^2^. Therefore, the identification of novel anticancer agents with higher potency and lower toxicity is needed for the treatment of aggressive and refractory cancers. More than 60% of all clinically used anticancer drugs originate from natural sources^3^, highlighting the success of natural products.

**Figure 1.**
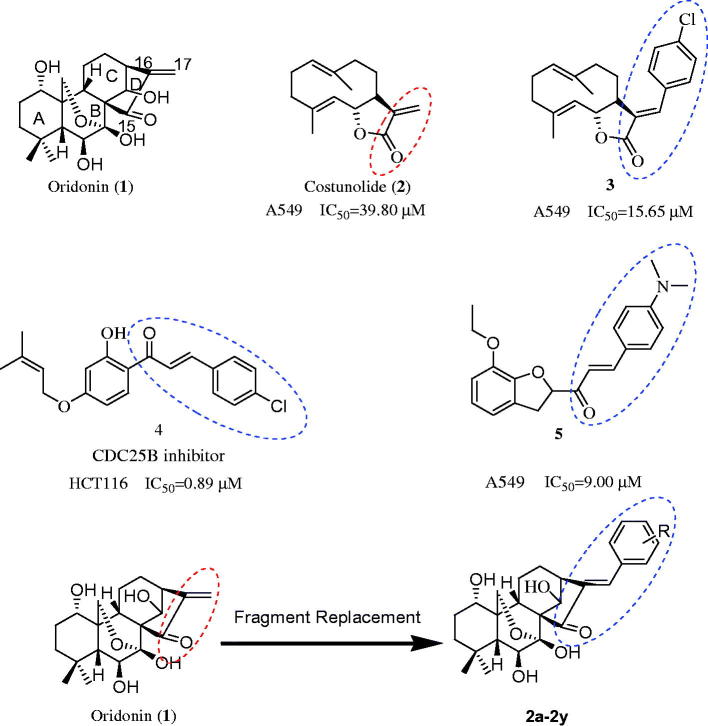
The structure of oridonin, costunolide, compound **3**, **4**, **5** and the design of target compound **2a–2y**.

Oridonin (**1**) (Figure 1) is an ent-kaurane diterpenoid isolated from *Isodon rubescens* (Donglingcao in Chinese). Since the identification of its antitumour potential in 1967, it has been used in traditional Chinese medicine^4^. It is reported to prevent hepatic fibrosis^5–7^, Alzheimer’s disease^8^, microbial infections^9^, and inflammation^10^. It has been shown to exert anticancer effects with limited adverse effects^11–17^. However, limited hydrophobic/hydrophilic partitioning and oral bioavailability diminish its therapeutic potential and clinical utility^18^. Therefore, it is necessary to develop novel oridonin analogues with potent antitumour efficacy through structural effective modifications.

Compounds with substituted benzene rings display a broad spectrum of biological activities; therefore, structural modification by adding substituted benzene rings is widely used in medicinal chemistry. It increases receptor binding by promoting hydrophobic interactions and enhances lipid solubility to facilitate drug permeation. In addition, an arylating *α,β*-unsaturated ketone system is commonly present in several anticancer agents, such as compounds **3–5** ^19–21^ (Figure 1). In a previous experiment, the arylated *α*,*β*-unsaturated ketone was added to the structure of costunolide; the resultant compound **3** was found to have better antiproliferative activity against A549 cells (IC_50 _= 15.65 µM) than costunolide (IC_50 _= 39.80 µM)^19^ (Figure 1). This substitution in the D ring was also found to be responsible for the antiproliferative activity of oridonin; the reduction or expansion of this ring is expected to significantly reduce efficacy. Inspired by these reports, we synthesised novel oridonin analogues, containing the arylated *α*,*β*-unsaturated ketone system in its D ring, to evaluate whether these analogues display better anticancer activity than oridonin. In this study, we designed, synthesised, and evaluated novel oridonin derivatives (**2a**–**2y**) containing a substituted benzene at the C-17 position (Figure 1) for their *in vitro* antitumour efficacy.

## Materials and methods

### Chemistry

IR spectra were recorded (in KBr) on IR Prestige-21. ^1^H-NMR and ^13^C-NMR spectra were measured on an AV-300 (Bruker BioSpin, Switzerland), ^13^C-NMR spectra were measured on an AV-500 (Bruker BioSpin, Switzerland), and all chemical shifts were given in ppm relative to tetramethylsilane (TMS). High-resolution mass spectra were measured using a matrix-assisted laser desorption/ionisation-time of flight (TOF)/TOF mass spectrometer (Bruker Daltonik, Bremen, Germany). The major chemicals were purchased from Aldrich Chemical Corporation. All other chemicals were of analytical grade.

### General procedure for the synthesis of compound (2a–2y)

To a stirred solution of oridonin (0.19 mmol, 70.00 mg), substituted iodobenzene (0.23 mmol) in N, N-dimethylformamide (5.00 ml), triethylamine (0.58 mmol, 58.18 mg), palladium acetate (0.01 mmol, 2.24 mg) and triphenylphosphine (0.020 mmol, 5.24 mg) was added. The reaction mixture was stirred at 90 °0 (oil bath temperature) for 20–24 h monitored by TLC and then concentrated under reduced pressure. The residue was added water (10.0 ml) and extracted with dichloromethane. The organic layer was washed with saturated NaHCO_3_, saturated NaCl and several portions of 15% hydrochloric acid solution, and dried over anhydrous Na_2_SO_4_. Then it can be purified by chromatography on silica eluting with a gradient of methanol/dichloromethane (1:80–1:30) to obtain the compounds (**2a–2y**) as white solids.

### (1*S*,4*aR*,5*S*,6*S*,6*aR*,9*S*,11*aS*,14*R,E*)-8-benzylidene-1,5,6,14-tetrahydroxy-4,4-dimethyldecahydro-1*H*-6,11b-(epoxymethano)-6a,9-methanocyclohepta[*a*]naphthale-n-7(8*H*)-one (2a)

Yield 70%. ^1^H-NMR (DMSO-d_6_, 300 MHz): *δ* 1.02 (s, 3H, –CH_3_), 1.04 (s, 3H, –CH_3_), 1.16–1.35 (m, 4H), 1.40–1.69 (m, 4H), 1.79–1.85 (m, 1H), 2.10–2.20 (m, 1H), 2.56–2.65 (m, 1H), 3.27–3.30 (m, 1H), 3.52–3.58 (m, 1H), 3.88 (d, 1H, *J* = 9.9 Hz), 4.15 (d, 1H, *J* = 9.6 Hz), 4.40 (d, 1H, *J* = 5.1 Hz), 4.86 (s, 1H), 6.03 (s, 1H), 6.24 (d, 1H, *J* = 10.5 Hz), 6.90 (s, 1H), 7.42 (s, 1H), 7.46–7.61 (m, 5H). ^13^C-NMR (DMSO-d_6_, 125 MHz): *δ* 19.89, 22.19, 28.44, 29.84, 33.21, 33.85, 38.87, 40.96, 41.37, 53.28, 60.04, 61.46, 63.25, 72.16, 73.47, 73.67, 97.54, 129.54, 130.35, 130.67, 133.19, 134.74, 143.63, 209.50. IR (KBr) cm^−1^: 3394, 2912, 1693, 1615. HRMS calcd. for C_26_H_32_NaO_6_ ([M + Na]^+^): 463.2091; found: 463.2085.

### (1*S*,4*aR*,5*S*,6*S*,6*aR*,9*S*,11*aS*,14*R,E*)-8-(3-fluorobenzylidene)-1,5,6,14-tetrahydroxy-4,4-dimethyldecahydro-1*H*-6,11b-(epoxymethano)-6a,9-methanocyclohepta[*a*]n-aphthalen-7(8*H*)-one (2b)

Yield 43%. ^1^H-NMR (DMSO-d_6_, 300 MHz): *δ* 1.01 (s, 3H, –CH_3_), 1.04 (s, 3H, –CH_3_), 1.15–1.35 (m, 4H), 1.43–1.67 (m, 4H), 1.79–1.83 (m, 1H), 2.12–2.20 (m, 1H), 2.57–2.67 (m, 1H), 3.27–3.30 (m, 1H), 3.52–3.58 (m, 1H), 3.88 (d, 1H, *J* = 9.9 Hz), 4.15 (d, 1H, *J* = 9.6 Hz), 4.41 (d, 1H, *J* = 5.1 Hz), 4.86 (s, 1H), 6.03 (s, 1H), 6.15 (d, 1H, *J* = 10.5 Hz), 6.91 (s, 1H), 7.29–7.59 (m, 5H). ^13^C-NMR (DMSO-d_6_, 125 MHz): *δ* 19.85, 22.17, 28.39, 29.81, 33.19, 33.84, 40.97, 41.33, 53.34, 60.01, 61.53, 63.27, 72.17, 73.39, 73.67, 97.50, 117.05, 126.56, 131.56, 137.15, 144.98, 161.77, 163.71, 209.43. IR (KBr) cm^−1^: 3404, 2902, 1695, 1618. HRMS calcd. for C_26_H_31_FNaO_6_ ([M + Na]^+^): 481.1997; found: 481.1989.

### (1*S*,4*aR*,5*S*,6*S*,6*aR*,9*S*,11*aS*,14*R*,*E*)-8-(4-fluorobenzylidene)-1,5,6,14-tetrahydroxy-4,4-dimethyldecahydro-1*H*-6,11b-(epoxymethano)-6a,9-methanocyclohepta[*a*]n-aphthalen-7(8*H*)-one (2c)

Yield 46%. ^1^H-NMR (DMSO-d_6_, 300 MHz): *δ* 1.01 (s, 3H, –CH_3_), 1.03 (s, 3H, –CH_3_), 1.14–1.34 (m, 4H), 1.43–1.66 (m, 4H), 1.80–1.82 (m, 1H), 2.12–2.20 (m, 1H), 2.57–2.67 (m, 1H), 3.27–3.30 (m, 1H), 3.50–3.56 (m, 1H), 3.88 (d, 1H, *J* = 9.9 Hz), 4.14 (d, 1H, *J* = 9.6 Hz), 4.41 (d, 1H, *J* = 5.1 Hz), 4.85 (s, 1H), 6.03 (s, 1H), 6.23 (d, 1H, *J* = 10.5 Hz), 6.91 (s, 1H), 7.31–7.37 (m, 2H), 7.42 (s, 1H), 7.64–7.70 (m, 2H). ^13^C-NMR (DMSO-d_6_, 125 MHz): *δ* 19.84, 22.15, 28.29, 29.79, 33.18, 33.83, 38.84, 40.95, 41.20, 53.26, 55.31, 56.52, 60.03, 61.42, 63.25, 72.18, 73.45, 73.65, 97.51, 116.71, 132.08, 133.06, 143.20, 162.23, 164.21, 209.44. IR (KBr) cm^−1^: 3395, 2900, 1693, 1625. HRMS calcd. for C_26_H_31_FNaO_6_ ([M + Na]^+^): 481.1997; found: 481.1987.

### (1*S*,4*aR*,5*S*,6*S*,6*aR*,9*S*,11*aS*,14*R*,*E*)-8-(3-chlorobenzylidene)-1,5,6,14-tetrahydrox-y-4,4-dimethyldecahydro-1*H*-6,11b-(epoxymethano)-6a,9-methanocyclohepta[*a*]-naphthalen-7(8*H*)-one (2d)

Yield 51%. ^1^H-NMR (DMSO-d_6_, 300 MHz): *δ* 1.01 (s, 3H, –CH_3_), 1.03 (s, 3H, –CH_3_), 1.14–1.34 (m, 4H), 1.51–1.67 (m, 4H), 1.79–1.85 (m, 1H), 2.12–2.20 (m, 1H), 2.51–2.65 (m, 1H), 3.27–3.30 (m, 1H), 3.51–3.57 (m, 1H), 3.88 (d, 1H, *J* = 9.9 Hz), 4.15 (d, 1H, *J* = 9.6 Hz), 4.41 (d, 1H, *J* = 5.1 Hz), 4.86 (s, 1H), 6.03 (s, 1H), 6.14 (d, 1H, *J* = 10.5 Hz), 6.89 (s, 1H), 7.40 (s, 1H), 7.52–7.54 (m, 2H), 7.63–7.64 (m, 2H) ^13^C-NMR (DMSO-d_6_, 125 MHz): *δ* 19.85, 22.17, 28.43, 29.81, 33.20, 33.84, 38.85, 40.96, 41.35, 53.34, 60.01, 61.54, 63.27, 72.16, 73.37, 73.66, 97.49, 128.73, 129.96, 130.24, 131.38, 131.55, 134.15, 136.93, 145.16, 209.38. IR (KBr) cm^−1^: 3450, 2925, 1689, 1621. HRMS calcd. for C_26_H_31_ClNaO_4_ ([M + Na]^+^): 497.1701; found: 497.1692.

### (1*S*,4*aR*,5*S*,6*S*,6*aR*,9*S*,11*aS*,14*R*,*E*)-8-(4-chlorobenzylidene)-1,5,6,14-tetrahydroxy-4,4-dimethyldecahydro-1*H*-6,11b-(epoxymethano)-6a,9-methanocyclohepta[*a*]-naphthalen-7(8*H*)-one (2e)

Yield 53%. ^1^H-NMR (DMSO-d_6_, 300 MHz): *δ* 1.01 (s, 3H, –CH_3_), 1.03 (s, 3H, –CH_3_), 1.14–1.39 (m, 4H), 1.43–1.66 (m, 4H), 1.78–1.82 (m, 1H), 2.12–2.20 (m, 1H), 2.57–2.67 (m, 1H), 3.27–3.30 (m, 1H), 3.51–3.57 (m, 1H), 3.87 (d, 1H, *J* = 9.9 Hz), 4.14 (d, 1H, *J* = 9.9 Hz), 4.41 (d, 1H, *J* = 5.1 Hz), 4.86 (s, 1H), 6.03 (s, 1H), 6.18 (d, 1H, *J* = 10.5 Hz), 6.90 (s, 1H), 7.40 (s, 1H), 7.54–7.63 (m, 4H). ^13^C-NMR (DMSO-d_6_, 125 MHz): *δ* 19.85, 22.16, 28.36, 29.80, 33.19, 33.83, 38.85, 40.96, 41.30, 53.31, 60.02, 61.48, 63.26, 72.17, 73.41, 73.66, 97.51, 129.60, 131.83, 132.32, 133.62, 134.94, 144.28, 209.41. IR (KBr) cm^−1^: 3441, 2932, 1685, 1615. HRMS calcd. for C_26_H_31_ClNaO_4_ ([M + Na]^+^): 497.1701; found: 497.1693.

### (1*S*,4*aR*,5*S*,6*S*,6*aR*,9*S*,11*aS*,14*R*,*E*)-1,5,6,14-tetrahydroxy-4,4-dimethyl-8-(2-methylbenzylidene)decahydro-1*H*-6,11b-(epoxymethano)-6a,9-methanocyclohepta[*a*]-naphthalen-7(8*H*)-one (2f)

Yield 74%. ^1^H-NMR (DMSO-d_6_, 300 MHz): *δ* 1.01 (s, 3H, –CH_3_), 1.03 (s, 3H, –CH_3_), 1.16–1.34 (m, 4H), 1.45–1.66 (m, 4H), 1.81–1.85 (m, 1H), 2.10–2.17 (m, 1H), 2.37 (s, 3H, Ar–CH_3_), 2.53–2.58 (m, 1H), 3.18 (d, 1H, *J* = 9.9 Hz), 3.52–3.55 (m, 1H), 3.87 (d, 1H, *J* = 9.9 Hz), 4.14 (d, 1H, *J* = 9.9 Hz), 4.38 (d, 1H, *J* = 5.1 Hz), 4.85 (s, 1H), 6.02 (s, 1H), 6.20 (d, 1H, *J* = 10.5 Hz), 6.85 (s, 1H), 7.32–7.34 (m, 3H), 7.39 (d, 1H, *J* = 7.0 Hz), 7.58 (s, 1H). ^13^C-NMR (DMSO-d_6_, 125 MHz): *δ* 19.93, 20.06, 22.15, 28.99, 29.83, 33.18, 33.86, 38.88, 40.94, 40.99, 53.42, 60.13, 61.64, 63.27, 72.15, 73.41, 73.63, 97.54, 126.80, 128.53, 130.09, 130.21, 131.20, 133.54, 138.87, 144.18, 209.55. IR (KBr) cm^−1^: 3405, 2925, 1674, 1632. HRMS calcd. for C_27_H_34_NaO_6_ ([M + Na]^+^): 477.2248; found: 477.2239.

### (1*S*,4*aR*,5*S*,6*S*,6*aR*,9*S*,11*aS*,14*R*,*E*)-1,5,6,14-tetrahydroxy-4,4-dimethyl-8-(3-methylbenzylidene)decahydro-1*H*-6,11b-(epoxymethano)-6a,9-methanocyclohepta[*a*]naphthalen-7(8*H*)-one (2g)

Yield 67%. ^1^H-NMR (DMSO-d_6_, 300 MHz): *δ* 1.01 (s, 3H, –CH_3_), 1.04 (s, 3H, –CH_3_), 1.17–1.31 (m, 4H), 1.44–1.67 (m, 4H), 1.78–1.83 (m, 1H), 2.12–2.21 (m, 1H), 2.37 (s, 3H), 2.59–2.65 (m, 1H), 3.27–3.30 (m, 1H), 3.52–3.57 (m, 1H), 3.87 (d, 1H, *J* = 9.9 Hz), 4.14 (d, 1H, *J* = 9.6 Hz), 4.40 (d, 1H, *J* = 5.1 Hz), 4.86 (s, 1H), 6.02 (s, 1H), 6.18 (d, 1H, *J* = 10.5 Hz), 6.90 (s, 1H), 7.29 (s, 1H), 7.39 (s, 4H). ^13^C-NMR (DMSO-d_6_, 125 MHz): *δ* 19.88, 21.45, 22.18, 28.46, 29.83, 33.20, 33.84, 38.86, 40.95, 41.39, 53.28, 55.28, 60.03, 63.25, 72.17, 73.46, 73.67, 97.54, 127.65, 129.43, 131.08, 131.34, 133.32, 134.69, 138.76, 143.46, 209.49. IR (KBr) cm^−1^: 3434, 2933, 1675, 1627. IR (KBr) cm^−1^: 3429, 2921, 1684, 1620. HRMS calcd. for C_27_H_34_NaO_6_ ([M + Na]^+^): 477.2248; found: 477.2236.

### (1*S*,4*aR*,5*S*,6*S*,6*aR*,9*S*,11*aS*,14*R*,*E*)-1,5,6,14-tetrahydroxy-4,4-dimethyl-8-(4-methylbenzylidene)decahydro-1*H*-6,11b-(epoxymethano)-6a,9-methanocyclohepta[*a*]naphthalen-7(8*H*)-one (2h)

Yield 78%. ^1^H-NMR (DMSO-d_6_, 300 MHz): *δ* 1.01 (s, 3H, –CH_3_), 1.03 (s, 3H, –CH_3_), 1.14–1.33 (m, 4H), 1.44–1.65 (m, 4H), 1.78–1.80 (m, 1H), 2.13–2.17 (m, 1H), 2.36 (s, 3H, Ar–CH_3_), 2.55–2.61 (m, 1H), 3.29–3.30 (m, 1H), 3.52–3.55 (m, 1H), 3.87 (d, 1H, *J* = 9.9 Hz), 4.14 (d, 1H, *J* = 9.9 Hz), 4.37 (d, 1H, *J* = 5.1 Hz), 4.84 (s, 1H), 6.00 (s, 1H), 6.27 (d, 1H, *J* = 10.5 Hz), 6.88 (s, 1H), 7.31 (d, 2H, *J* = 7.0 Hz), 7.37 (s, 1H), 7.49 (d, 2H, *J* = 7.0 Hz). ^13^C-NMR (DMSO-d_6_, 125 MHz): *δ* 19.88, 21.52, 22.19, 28.31, 29.84, 33.21, 33.85, 38.87, 40.95, 41.38, 53.23, 60.04, 61.39, 63.24, 72.17, 73.49, 73.67, 97.55, 130.18, 130.74, 131.97, 133.32, 140.43, 142.55, 209.44. IR (KBr) cm^−1^: 3425, 2925, 1678, 1615. HRMS calcd. for C_27_H_34_NaO_6_ ([M + Na]^+^): 477.2248; found: 477.2238.

### (1*S,*4*aR*,5*S*,6*S*,6*aR*,9*S*,11*aS*,14*R*,*E*)-8-(3,4-dimethylbenzylidene)-1,5,6,14-tetrahydroxy-4,4-dimethyldecahydro-1*H*-6,11b-(epoxymethano)-6a,9-methanocyclohept-a-[*a*]naphthalen-7(8*H*)-one (2i)

Yield 72%. ^1^H-NMR (DMSO-d_6_, 300 MHz): *δ* 1.01 (s, 3H, –CH_3_), 1.04 (s, 3H, –CH_3_), 1.14–1.34 (m, 4H), 1.43–1.64 (m, 4H), 1.76–1.80 (m, 1H), 2.12–2.19 (m, 1H), 2.27 (s, 6H), 2.57–2.65 (m, 1H), 3.27–3.30 (m, 1H), 3.51–3.57 (m, 1H), 3.87 (d, 1H, *J* = 9.9 Hz), 4.14 (d, 1H, *J* = 9.6 Hz), 4.39 (d, 1H, *J* = 5.1 Hz), 4.84 (s, 1H), 6.00 (s, 1H), 6.30 (d, 1H, *J* = 10.5 Hz), 6.90 (s, 1H), 7.25–7.36 (m, 4H). ^13^C-NMR (DMSO-d_6_, 125 MHz): *δ* 19.88, 19.91, 22.19, 28.35, 29.84, 33.22, 33.85, 33.88, 40.95, 41.40, 53.23, 55.38, 60.04, 61.38, 63.23, 72.16, 73.48, 73.67, 97.56, 128.14, 130.67, 131.92, 132.34, 133.48, 137.47, 139.34, 142.41, 209.42. IR (KBr) cm^−1^: 3452, 2935, 1669, 1612. HRMS calcd. for C_28_H_36_NaO_6_ ([M + Na]^+^): 491.2404; found: 491.2396.

### (1*S*,4*aR*,5*S*,6*S*,6*aR*,9*S*,11*aS*,14*R*,*E*)-1,5,6,14-tetrahydroxy-8-(2-methoxybenzylidene)-4,4-dimethyldecahydro-1*H*-6,11b-(epoxymethano)-6a,9-methanocyclohepta-[*a*]naphthalen-7(8*H*)-one (2j)

Yield 78%. ^1^H-NMR (DMSO-d_6_, 300 MHz): *δ* 1.01 (s, 3H, –CH_3_), 1.04 (s, 3H, –CH_3_), 1.15–1.34 (m, 4H), 1.43–1.65 (m, 4H), 1.79–1.85 (m, 1H), 2.11–2.19 (m, 1H), 2.57–2.63 (m, 1H), 3.19–3.22 (m, 1H), 3.48–3.55 (m, 1H), 3.81–3.92 (m, 4H), 4.14 (d, 1H, *J* = 9.6 Hz), 4.40 (d, 1H, *J* = 5.1 Hz), 4.84 (s, 1H), 6.01 (s, 1H), 6.27 (d, 1H, *J* = 10.5 Hz), 6.87 (s, 1H), 7.06–7.13 (m, 2H), 7.40–7.45 (m, 2H), 7.72 (s, 1H). ^13^C-NMR (DMSO-d_6_, 125 MHz): *δ* 19.84, 22.15, 28.29, 29.79, 33.18, 33.83, 38.84, 40.95, 41.20, 53.26, 56.53, 60.03, 61.42, 72.18, 73.45, 73.65, 97.51, 116.71, 131.34, 132.99, 133.06, 143.20, 162.23, 164.21, 209.44. IR (KBr) cm^−1^: 3437, 2928, 1673, 1622. HRMS calcd. for C_27_H_34_NaO_7_ ([M + Na]^+^): 493.2197; found: 493.2190.

### (1*S*,4*aR*,5*S*,6*S*,6*aR*,9*S*,11*aS*,14*R*,*E*)-1,5,6,14-tetrahydroxy-8-(4-methoxybenzylidene)-4,4-dimethyldecahydro-1*H*-6,11b-(epoxymethano)-6a,9-methanocyclohepta-[*a*]naphthalen-7(8*H*)-one (2k)

Yield 83%. ^1^H-NMR (DMSO-d_6_, 300 MHz): *δ* 1.00 (s, 3H, –CH_3_), 1.03 (s, 3H, –CH_3_), 1.14–1.34 (m, 4H), 1.44–1.65 (m, 4H), 1.76–1.81 (m, 1H), 2.13–2.22 (m, 1H), 2.55–2.63 (m, 1H), 3.26–3.29 (m, 1H), 3.50–3.55 (m, 1H), 3.82 (s, 3H), 3.87 (d, 1H, *J* = 9.9 Hz), 4.13 (d, 1H, *J* = 9.6 Hz), 4.40 (d, 1H, *J* = 5.1 Hz), 4.84 (s, 1H), 6.02 (s, 1H), 6.38 (d, 1H, *J* = 10.5 Hz), 6.90 (s, 1H), 7.07 (d, 2H, *J* = 8.4 Hz), 7.37 (s, 1H), 7.57 (d, 2H, *J* = 8.4 Hz). ^13^C-NMR (DMSO-d_6_, 125 MHz): 19.86, 22.13, 28.13, 29.78, 33.17, 33.82, 38.84, 40.94, 41.31, 53.15, 56.54, 60.07, 61.28, 63.21, 72.19, 73.57, 73.64, 97.55, 115.13, 127.21, 132.67, 133.37, 140.77, 161.16, 209.34. IR (KBr) cm^−1^: 3430, 2924, 1670, 1626. HRMS calcd. for C_27_H_34_NaO_7_ ([M + Na]^+^): 493.2197; found: 493.2189.

### (1*S*,4*aR*,5*S*,6*S*,6*aR*,9*S*,11*aS*,14*R*,*E*)-8-(2,4-dimethoxybenzylidene)-1,5,6,14-tetrahydroxy-4,4-dimethyldecahydro-1*H*-6,11b-(epoxymethano)-6a,9-methanocyclohe-pta[*a*]naphthalen-7(8*H*)-one (2l)

Yield 82%. ^1^H-NMR (DMSO-d_6_, 300 MHz): *δ* 1.01 (s, 3H, –CH_3_), 1.03 (s, 3H, –CH_3_), 1.14–1.34 (m, 4H), 1.44–1.65 (m, 4H), 1.75–1.79 (m, 1H), 2.08–2.20 (m, 1H), 2.55–2.61 (m, 1H), 3.26–3.29 (m, 1H), 3.49–3.55 (m, 1H), 3.84 (s, 3H), 3.87 (s, 3H), 4.10–4.14 (m, 1H), 4.37 (d, 1H, *J* = 5.1 Hz), 4.83 (s, 1H), 5.98 (s, 1H), 6.40 (d, 1H, *J* = 10.5 Hz), 6.66 (s, 2H), 66.86 (s, 1H), 7.40 (d, 2H, *J* = 8.4 Hz), 7.69 (s, 1H). ^13^C-NMR (DMSO-d_6_, 125 MHz): *δ* 19.92, 22.16, 28.29, 29.85, 33.19, 33.85, 38.89, 40.92, 41.23, 53.15, 56.00, 56.35, 60.14, 61.30, 63.22, 72.16, 73.62, 97.60, 106.62, 116.09, 127.30, 130.60, 140.29, 160.58, 163.00, 209.32. IR (KBr) cm^−1^: 3445, 2913, 1682, 1628. HRMS calcd. for C_28_H_36_NaO_8_ ([M + Na]^+^): 523.2302; found: 493.2295.

### (1*S*,4*aR*,5*S*,6*S*,6*aR*,9*S*,11*aS*,14*R*,*E*)-8-(3,4-dimethoxybenzylidene)-1,5,6,14-tetrahydroxy-4,4-dimethyldecahydro-1*H*-6,11b-(epoxymethano)-6a,9-methanocyclohepta[*a*]naphthalen-7(8*H*)-one (2m)

Yield 85%. ^1^H-NMR (DMSO-d_6_, 300 MHz): *δ* 1.01 (s, 3H, –CH_3_), 1.04 (s, 3H, –CH_3_), 1.14–1.34 (m, 4H), 1.43–1.67 (m, 4H), 1.76–1.80 (m, 1H), 2.08–2.21 (m, 1H), 2.51–2.59 (m, 1H), 3.18 (d, *J* = 5.4 Hz, 1H), 3.26–3.29 (m, 1H), 3.51–3.57 (m, 1H), 3.81 (s, 3H, -OCH_3_), 3.82 (s, 3H, -OCH_3_), 3.87 (d, 1H, *J* = 9.9 Hz), 4.10–4.16 (m, 1H), 4.38 (d, 1H, *J* = 5.1 Hz), 4.85 (s, 1H), 6.00 (s, 1H), 6.91 (d, 1H, *J* = 10.5 Hz), 7.08–7.21 (m, 3H), 7.38 (s, 1H).^13^C-NMR (DMSO-d_6_, 125 MHz): *δ* 19.89, 22.19, 28.11, 29.84, 33.21, 33.84, 38.87, 40.94, 41.40, 53.17, 55.35, 55.91, 56.09, 60.04, 63.22, 72.18, 73.54, 73.67, 97.58, 112.44, 113.72, 124.48, 127.45, 133.75, 140.95, 149.24, 150.96, 209.21. IR (KBr) cm^−1^: 3440, 2923, 1673, 1632. HRMS calcd. for C_28_H_36_NaO_8_ ([M + Na]^+^): 523.2302; found: 493.2296.

### (1*S*,4*aR*,5*S*,6*S*,6*aR*,9*S*,11*aS*,14*R*,*E*)-1,5,6,14-tetrahydroxy-4,4-dimethyl-8-(3,4,5-trimethoxybenzylidene)decahydro-1*H*-6,11b-(epoxymethano)-6a,9-methanocyclo-hepta[*a*]naphthalen-7(8*H*)-one (2n)

Yield 87%. ^1^H-NMR (DMSO-d_6_, 300 MHz): *δ* 1.02 (s, 3H, –CH_3_), 1.04 (s, 3H, –CH_3_), 1.15–1.35 (m, 4H), 1.47–1.64 (m, 4H), 1.78–1.82 (m, 1H), 2.08–2.19 (m, 1H), 2.55–2.63 (m, 1H), 3.35–3.37 (m, 1H), 3.52–3.57 (m, 1H), 3.72 (s, 3H, -OCH_3_), 3.83 (s, 6H, -OCH_3_), 3.89 (d, 1H, *J* = 9.9 Hz), 4.14 (d, 1H, *J* = 9.6 Hz), 4.39 (d, 1H, *J* = 5.1 Hz), 4.85 (s, 1H), 5.98 (s, 1H), 6.28 (d, 1H, *J* = 10.5 Hz), 6.90 (s, 2H), 6.93 (s, 1H), 7.38 (s, 1H). ^13^C-NMR (DMSO-d_6_, 125 MHz): *δ* 19.88, 22.21, 28.21, 29.84, 33.23, 33.85, 38.86, 40.95, 41.45, 53.24, 56.38, 59.97, 60.04, 61.42, 63.25, 72.17, 73.46, 73.68, 97.56, 108.10, 130.23, 133.62, 139.57, 142.60, 153.46, 209.29. IR (KBr) cm^−1^: 3390, 2923, 1675, 1616. HRMS calcd. for C_29_H_38_NaO_9_ ([M + Na]^+^): 553.2408; found: 553.2398.

### (1*S*,4*aR*,5*S*,6*S*,6*aR*,9*S*,11*aS*,14*R*,*E*)-1,5,6,14-tetrahydroxy-4,4-dimethyl-8-(4-propoxybenzylidene)decahydro-*1H*-6,11b-(epoxymethano)-6a,9-methanocyclohepta-[*a*]naphthalen-7(8*H*)-one (2o)

Yield 85%. ^1^H-NMR (DMSO-d_6_, 300 MHz): *δ* 0.98–1.03 (m, 9H, –CH_3_), 1.15–1.34 (m, 6H), 1.47–1.64 (m, 4H), 1.73–1.78 (m, 2H), 2.08–2.20 (m, 1H), 2.57–2.65 (m, 1H), 3.25–3.28 (m, 1H), 3.50–3.55 (m, 1H), 3.87 (d, 1H, *J* = 9.9 Hz), 4.00 (t, 2H, *J* = 6.0 Hz), 4.13 (d, 1H, *J* = 9.6 Hz), 4.40 (d, 1H, *J* = 5.1 Hz), 4.83 (s, 1H), 6.02 (s, 1H), 6.37 (d, 1H, *J* = 10.5 Hz), 6.90 (s, 1H), 7.05 (d, 2H, *J* = 8.1 Hz), 7.55 (d, 2H, *J* = 8.1 Hz). ^13^C-NMR (DMSO-d_6_, 125 MHz): *δ* 10.80, 19.01, 19.88, 22.18, 22.40, 28.14, 29.84, 33.21, 33.84, 40.95, 41.31, 53.16, 56.51, 60.09, 61.30, 63.22, 69.66, 72.19, 73.66, 97.58, 115.56, 127.12, 132.66, 133.34, 140.74, 160.62, 209.28. IR (KBr) cm^−1^: 3341, 2936, 1678, 1626. HRMS calcd. for C_29_H_38_NaO_7_ ([M + Na]^+^): 521.2510; found: 521.2501.

### (1*S*,4*aR*,5*S*,6*S*,6*aR*,9*S*,11*aS*,14*R*,*E*)-1,5,6,14-tetrahydroxy-4,4-dimethyl-8-(4-((3-methylbut-2-en1yl)oxy)benzylidene)decahydro-1*H*-6,11b-(epoxymethano)-6a,9-methanocyclohepta[*a*]naphthalen-7(8*H*)-one (2p)

Yield 72%. ^1^H-NMR (DMSO-d_6_, 300 MHz): *δ* 1.00 (s, 3H, –CH_3_), 1.03 (s, 3H, –CH_3_), 1.13–1.29 (m, 4H), 1.43–1.63 (m, 4H), 1.72 (s, 3H, –CH_3_), 1.75 (s, 3H, –CH_3_), 1.79–1.83 (m, 1H), 2.07–2.25 (m, 1H), 2.55–2.66 (m, 1H), 3.25–3.28 (m, 1H), 3.50–3.55 (m, 1H), 3.86 (d, 1H, *J* = 9.9 Hz), 4.13 (d, 1H, *J* = 9.6 Hz), 4.40 (d, 1H, *J* = 5.1 Hz), 4.59 (d, 2H, *J* = 6.3 Hz, –OCH_2_–) 4.83 (s, 1H), 5.44 (t, 1H, *J* = 6.0 Hz), 6.02 (s, 1H), 6.37 (d, 1H, *J* = 10.5 Hz), 6.90 (s, 1H), 7.05 (d, 2H, *J* = 8.4 Hz), 7.37 (s, 1H), 7.54 (d, 2H, *J* = 8.4 Hz). ^13^C-NMR (DMSO-d_6_, 125 MHz): *δ* 18.51, 19.01, 19.89, 22.18, 25.89, 28.14, 29.84, 33.21, 33.84, 38.89, 40.94, 41.30, 53.16, 56.51, 60.09, 61.30, 63.22, 65.02, 72.19, 73.66, 97.58, 115.74, 116.99, 127.14, 132.61, 138.11, 140.75, 160.40, 209.29. IR (KBr) cm^−1^: 3343, 2927, 1674, 1628. HRMS calcd. for C_31_H_40_NaO_7_ ([M + Na]^+^): 547.2666; found: 547.2655.

### (1*S*,4*aR*,5*S*,6*S*,6*aR*,9*S*,11*aS*,14*R*,*E*)-1,5,6,14-tetrahydroxy-4,4-dimethyl-8-(3-((3-methylbut-2-en1yl)oxy)benzylidene)decahydro-1*H*-6,11b-(epoxymethano)-6a,9-methanocyclohepta[*a*]naphthalen-7(8*H*)-one (2q)

Yield 65%. ^1^H-NMR (DMSO-d_6_, 300 MHz): *δ* 1.02 (s, 3H, –CH_3_), 1.04 (s, 3H, –CH_3_), 1.15–1.30 (m, 4H), 1.44–1.61 (m, 5H), 1.72 (s, 3H, –CH_3_), 1.76 (s, 3H, –CH_3_), 1.81–1.84 (m, 1H), 2.08–2.20 (m, 1H), 2.55–2.67 (m, 1H), 3.26–3.29 (m, 1H), 3.52–3.58 (m, 1H), 3.88 (d, 1H, *J* = 9.9 Hz), 4.14 (d, 1H, *J* = 9.6 Hz), 4.40 (d, 1H, *J* = 5.1 Hz), 4.58 (d, 2H, *J* = 6.3 Hz, –OCH_2_–) 4.86 (s, 1H), 5.46 (t, 1H, *J* = 6.0 Hz), 6.02 (s, 1H), 6.23 (d, 1H, *J* = 10.5 Hz), 6.90 (s, 1H), 7.02–7.05 (m, 1H), 7.13–7.17 (m, 2H), 7.39–7.40 (m, 1H). ^13^C-NMR (DMSO-d_6_, 125 MHz): *δ* 18.54, 19.89, 22.19, 25.89, 28.44, 29.83, 33.21, 33.85, 38.87, 40.96, 41.44, 53.27, 60.02, 61.47, 63.25, 64.90, 72.16, 73.45, 73.67, 97.53, 116.54, 116.93, 120.26, 122.70, 130.55, 133.25, 136.05, 137.74, 143.78, 159.16, 209.47. IR (KBr) cm^−1^: 3333, 2924, 1680, 1625. HRMS calcd. for C_31_H_40_NaO_7_ ([M + Na]^+^): 547.2666; found: 547.2657.

### (1*S*,4*aR*,5*S*,6*S*,6*aR*,9*S*,11*aS*,14*R*,*E*)-8-(4-(((*Z*)-3,6-dimethylhepta-2,5-dien1yl)oxy)benzylidene)-1,5,6,14-tetrahydroxy-4,4-dimethyldecahydro-1*H*-6,11b-(epoxymethano)-6a,9-methanocyclohepta[*a*]naphthalen-7(8*H*)-one (2r)

Yield 79%. ^1^H-NMR (DMSO-d_6_, 300 MHz): *δ* 1.01 (s, 3H, –CH_3_), 1.04 (s, 3H, –CH_3_), 1.14–1.30 (m, 5H), 1.43–1.53 (m, 3H), 1.57 (s, 3H), 1.63 (s, 3H), 1.67 (s, 1H), 1.72 (s, 3H, –CH_3_), 1.76–1.81 (m, 1H), 2.03–2.20 (m, 5H), 2.26–2.29 (m, 1H), 2.51–1.62 (m, 1H), 3.51–3.56 (m, 1H), 3.87 (d, 1H, *J* = 9.9 Hz), 4.14 (d, 1H, *J* = 9.6 Hz), 4.38 (d, 1H, *J* = 5.1 Hz), 4.62 (d, 2H, *J* = 6.3 Hz, –OCH_2_–) 4.84 (s, 1H), 5.07 (t, 1H, *J* = 6.0 Hz), 5.43 (t, 1H, *J* = 6.0 Hz), 6.01 (s, 1H), 6.37 (d, 1H, *J* = 10.5 Hz), 6.89 (s, 1H), 7.05 (d, 2H, *J* = 9.0 Hz), 7.37 (s, 1H), 7.55 (d, 2H, *J* = 9.0 Hz). ^13^C-NMR (DMSO-d_6_, 125 MHz): *δ* 16.84, 18.03, 19.58, 22.18, 22.63, 25.94, 26.25, 28.14, 29.84, 33.21, 33.84, 38.88, 40.94, 41.30, 53.16, 60.08, 61.30, 63.22, 65.07, 72.18, 73.66, 97.58, 110.69, 115.77, 119.80, 124.21, 127.41, 131.52, 132.60, 133.35, 140.74, 141.18, 160.39, 209.29. IR (KBr) cm^−1^: 3349, 2934, 1670, 1623. HRMS calcd. for C_36_H_49_NaO_7_([M + Na]^+^): 615.3292; found: 615.3286.

### (1*S*,4*aR*,5*S*,6*S*,6*aR*,9*S*,11*aS*,14*R*,*E*)-8-(4-aminobenzylidene)-1,5,6,14-tetrahydroxy-4,4-dimethyldecahydro-1*H*-6,11b-(epoxymethano)-6a,9-methanocyclohepta[*a*]-naphthalen-7(8*H*)-one (2s)

Yield 70%. ^1^H-NMR (DMSO-d_6_, 300 MHz): *δ* 1.01 (s, 3H, –CH_3_), 1.02 (s, 3H, –CH_3_), 1.12–1.33 (m, 4H), 1.42–1.62 (m, 4H), 1.74–1.80 (m, 1H), 2.07–2.22 (m, 1H), 3.17 (s, 2H), 3.22–3.27 (m, 1H), 3.48–3.55 (m, 1H), 3.85 (d, 1H, *J* = 9.9 Hz), 4.12 (d, 1H, *J* = 9.6 Hz), 4.38 (d, 1H, *J* = 5.1 Hz), 4.81 (s, 1H), 5.98 (s, 1H), 6.43 (d, 1H, *J* = 9.9 Hz), 6.80 (d, 1H, *J* = 8.4 Hz), 6.90 (s, 1H), 7.20 (d, 1H, *J* = 8.7 Hz), 7.30 (d, 1H, *J* = 8.7 Hz), 7.37–7.48 (m, 2H). ^13^C-NMR (DMSO-d_6_, 125 MHz): *δ* 19.90, 22.16, 27.89, 29.85, 33.84, 38.90, 40.92, 41.42, 53.02, 55.35, 56.51, 60.16, 61.11, 63.18, 72.19, 73.64, 97.65, 113.56, 122.72, 132.79, 134.78, 137.32, 149.84, 208.86. IR (KBr) cm^−1^: 3345, 2934, 1667, 1621. HRMS calcd. for C_26_H_33_NNaO_6_ ([M + Na]^+^): 478.2200; found: 478.2192.

### *N*-(4-((*E*)-((1*S*,4*aR*,5*S*,6*S*,6*aR*,9*S*,11*aS*,14*R*)-1,5,6,14-tetrahydroxy-4,4-dimethyl-7-oxodecahydro-1*H*-6,11b-(epoxymethano)-6a,9-methanocyclohepta[*a*]naphthalen-8(7*H*)-ylidene)methyl)phenyl)acetamide (2t)

Yield 63%. ^1^H-NMR (DMSO-d_6_, 300 MHz): *δ* 1.00 (s, 3H, –CH_3_), 1.03 (s, 3H, –CH_3_), 1.15–1.29 (m, 4H), 1.43–1.67 (m, 4H), 1.78–1.82 (m, 1H), 1.99 (s, 1H), 2.08 (s, 3H), 2.57–2.66 (m, 1H), 3.53–3.55 (m, 1H), 3.87 (d, 1H, *J* = 9.9 Hz), 4.04 (q, 1H), 4.14 (d, 1H, *J* = 9.6 Hz), 4.40 (d, 1H, *J* = 5.1 Hz), 4.83 (s, 1H), 6.02 (s, 1H), 6.30 (d, 1H, *J* = 10.5 Hz), 6.91 (s, 1H), 7.33 (s, 1H), 7.55 (d, 2H, *J* = 8.4 Hz), 7.70 (d, 2H, *J* = 8.4 Hz), 10.23 (s, 1H, –CONH–). ^13^C-NMR (DMSO-d_6_, 125 MHz): *δ* 19.00, 19.91, 22.14, 28.63, 29.82, 33.16, 33.86, 38.86, 40.93, 41.16, 53.29, 56.18, 60.11, 63.26, 72.16, 73.62, 97.54, 112.04, 121.15, 123.25, 127.27, 129.37, 132.13, 143.10, 158.88, 209.57. IR (KBr) cm^−1^: 3351, 2940, 1668, 1619. HRMS calcd. for C_28_H_35_NNaO_7_ ([M + Na]^+^): 520.2306; found: 520.2297.

### *N*-(4-((*E*)-((1*S*,4*aR*,5*S*,6*S*,6*aR*,9*S*,11*aS*,14*R*)-1,5,6,14-tetrahydroxy-4,4-dimethyl-7-oxodecahydro-1*H*-6,11b-(epoxymethano)-6a,9-methanocyclohepta[*a*]naphthalen-8(7*H*)-ylidene)methyl)phenyl)benzamide (2u)

Yield 65%. ^1^H-NMR (DMSO-d_6_, 300 MHz): *δ* 1.02 (s, 3H, –CH_3_), 1.04 (s, 3H, –CH_3_), 1.15–1.35 (m, 4H), 1.44–1.66 (m, 4H), 1.78–1.82 (m, 1H), 2.11–2.21 (m, 1H), 2.57–2.68 (m, 1H), 3.26–3.30 (m, 1H), 3.52–3.58 (m, 1H), 3.88 (d, 1H, *J* = 9.9 Hz), 4.15 (d, 1H, *J* = 9.6 Hz), 4.40 (d, 1H, *J* = 5.1 Hz), 4.86 (s, 1H), 6.04 (s, 1H), 6.33 (d, 1H, *J* = 10.5 Hz), 6.92 (s, 1H), 7.39 (s, 1H), 7.53–7.63 (m, 5H), 7.96 (t, 4H, *J* = 8.7 Hz), .10.51 (s, 1H, –CONH–). ^13^C-NMR (DMSO-d_6_, 125 MHz): *δ* 19.89, 22.19, 28.25, 29.85, 33.21, 33.85, 38.88, 40.96, 41.39, 53.25, 60.07, 61.38, 63.24, 72.18, 73.54, 73.68, 97.58, 120.79, 128.23, 128.92, 129.88, 131.57, 131.85, 132.28, 133.07, 141.27, 141.99, 166.34, 209.40. IR (KBr) cm^−1^: 3343, 2949, 1666, 1621. HRMS calcd. for C_33_H_37_NNaO_7_ ([M + Na]^+^): 582.2462; found: 582.2458.

### (1*S*,4*aR*,5*S*,6*S*,6*aR*,9*S*,11*aS*,14*R*,*E*)-1,5,6,14-tetrahydroxy-8-(2-hydroxybenzylidene)-4,4-dimethyldecahydro-1*H*-6,11b-(epoxymethano)-6a,9-methanocyclohepta[*a*]naphthalen-7(8*H*)-one (2v)

Yield 79%. ^1^H-NMR (DMSO-d_6_, 300 MHz): *δ* 1.01 (s, 3H, –CH_3_), 1.03 (s, 3H, –CH_3_), 1.14–1.35 (m, 4H), 1.44–1.63 (m, 4H), 1.77–1.87 (m, 1H), 2.06–2.21 (m, 1H), 3.20–3.26 (m, 1H), 3.49–3.58 (m, 1H), 3.86 (d, 1H, *J* = 9.9 Hz), 4.13 (d, 1H, *J* = 9.6 Hz), 4.41 (d, 1H, *J* = 5.1 Hz), 4.84 (s, 1H), 6.02 (s, 1H), 6.34 (d, 1H, *J* = 10.5 Hz), 6.88–6.95 (m, 3H), 7.27–7.38 (m, 2H), 7.76 (s, 2H), 10.23 (s, 1H, –ArOH). ^13^C-NMR (DMSO-d_6_, 125 MHz): *δ* 19.02, 22.16, 28.52, 29.84, 33.19, 33.85, 38.89, 40.94, 41.19, 53.24, 56.50, 60.13, 61.37, 63.24, 72.17, 73.64, 97.58, 116.38, 119.88, 121.74, 127.95, 129.33, 131.94, 141.85, 157.95, 209.55. IR (KBr) cm^−1^: 3356, 2943, 1672, 1617. HRMS calcd. for C_26_H_32_NaO_7_ ([M + Na]^+^): 479.2040; found: 479.2031.

### (1*S*,4*aR*,5*S*,6*S*,6*aR*,9*S*,11*aS*,14*R*,*E*)-1,5,6,14-tetrahydroxy-8-(3-hydroxybenzylidene)-4,4-dimethyldecahydro-1*H*-6,11b-(epoxymethano)-6a,9-methanocyclohepta[*a*]naphthalen-7(8*H*)-one (2w)

Yield 66%. ^1^H-NMR (DMSO-d_6_, 300 MHz): *δ* 1.01 (s, 3H, –CH_3_), 1.03 (s, 3H, –CH_3_), 1.14–1.34 (m, 4H), 1.43–1.66 (m, 4H), 1.78–1.82 (m, 1H), 2.09–2.26 (m, 1H), 2.57–2.64 (m, 1H), 3.26–3.29 (m, 1H), 3.50–3.56 (m, 1H), 3.86 (d, 1H, *J* = 9.9 Hz), 4.13 (d, 1H, *J* = 9.6 Hz), 4.41 (d, 1H, *J* = 5.1 Hz), 4.85 (s, 1H), 6.04 (s, 1H), 6.25 (d, 1H, *J* = 10.5 Hz), 6.84–6.88 (m, 2H), 6.99–7.03 (m, 2H), 7.26–7.31 (m, 2H), 9.72 (s, 1H, –ArOH). ^13^C-NMR (DMSO-d_6_, 125 MHz): *δ* 19.88, 22.17, 28.50, 29.83, 33.20, 33.84, 38.87, 40.95, 41.45, 53.26, 60.04, 61.44, 63.25, 72.17, 73.47, 73.65, 97.53, 116.69, 117.63, 122.02, 130.52, 133.42, 135.90, 143.35, 158.13, 209.50. IR (KBr) cm^−1^: 3339, 2945, 1668, 1620. HRMS calcd. for C_26_H_32_NaO_7_ ([M + Na]^+^): 479.2040; found: 479.2032.

### (1*S*,4*aR*,5*S*,6*S*,6*aR*,9*S*,11*aS*,14*R*,*E*)-1,5,6,14-tetrahydroxy-8-(4-hydroxybenzylidene)-4,4-dimethyldecahydro-1*H*-6,11b-(epoxymethano)-6a,9-methanocyclohepta[*a*]naphthalen-7(8*H*)-one (2x)

Yield 80%. ^1^H-NMR (DMSO-d_6_, 300 MHz): *δ* 1.01 (s, 3H, –CH_3_), 1.03 (s, 3H, –CH_3_), 1.14–1.39 (m, 4H), 1.43–1.65 (m, 4H), 1.75–1.79 (m, 1H), 2.04–2.26 (m, 1H), 2.56–2.62 (m, 1H), 3.24–3.27 (m, 1H), 3.50–3.55 (m, 1H), 3.86 (d, 1H, *J* = 9.9 Hz), 4.13 (d, 1H, *J* = 9.6 Hz), 4.39 (d, 1H, *J* = 5.1 Hz), 4.83 (s, 1H), 6.01 (s, 1H), 6.42 (d, 1H, *J* = 10.5 Hz), 6.88 (m, 3H), 7.33 (s, 1H), 7.47 (d, 2H, *J* = 7.5 Hz), 10.15 (s, 1H, –ArOH). ^13^C-NMR (DMSO-d_6_, 125 MHz): *δ* 18.97, 19.84, 22.21, 27.99, 29.79, 33.21, 33.77, 40.89, 41.26, 53.06, 56.48, 60.05, 61.22, 63.17, 72.12, 73.67, 97.58, 116.52, 125.71, 132.93, 133.81, 139.72, 159.92, 209.26. IR (KBr) cm^−1^: 3339, 2945, 1668, 1620. HRMS calcd. for C_26_H_32_NaO_7_ ([M + Na]^+^): 479.2040; found: 479.2031.

### (1*S*,4*aR*,5*S*,6*S*,6*aR*,9S,11*aS*,14*R*,*E*)-1,5,6,14-tetrahydroxy-4,4-dimethyl-8-(napht-halen-2-ylmethylene)decahydro-1*H*-6,11b-(epoxymethano)-6a,9-methanocyclohepta[*a*]naphthalen-7(8*H*)-one (2y)

Yield 67%. ^1^H-NMR (DMSO-d_6_, 300 MHz): *δ* 1.03 (s, 3H, –CH_3_), 1.04 (s, 3H, –CH_3_), 1.19–1.35 (m, 4H), 1.44–1.66 (m, 4H), 1.87–1.91 (m, 1H), 2.08–2.23 (m, 1H), 2.55–2.63 (m, 1H), 3.19–3.23 (m, 1H), 3.53–3.58 (m, 1H), 3.88 (d, 1H, *J* = 9.9 Hz), 4.15 (d, 1H, *J* = 9.6 Hz), 4.43 (d, 1H, *J* = 5.1 Hz), 4.88 (s, 1H), 6.07 (s, 1H), 6.26 (d, 1H, *J* = 10.5 Hz), 6.90 (s, 1H), 7.62–7.64 (m, 4H), 8.01–8.05 (m, 2H), 8.14 (s, 2H). ^13^C-NMR (DMSO-d_6_, 125 MHz): *δ* 19.00, 19.95, 22.13, 29.18, 29.82, 33.16, 33.87, 38.87, 40.97, 41.29, 53.53, 56.51, 60.17, 61.89, 63.30, 72.17, 74.65, 97.56, 123.97, 126.09, 126.95, 127.26, 127.63, 129.28, 130.49, 131.38, 131.98, 133.74, 145.85, 209.36. IR (KBr) cm^−1^: 3332, 2937, 1668, 1625. HRMS calcd. for C_30_H_34_NaO_6_ ([M + Na]^+^): 513.2248; found: 513.2240.

## Biological evaluation

### Materials

3-[4,5-Dimethylthiazol-2-yl]-2,5-diphenyl-tetrazolium bromide (MTT) was purchased from Sigma Chemical Co. (St. Louis, MO). The propidium iodide (PI) and Annexin V-FITC apoptosis detection kit was purchased from BD Pharmingen (SanDiego, CA).

### Cell lines and cell culture

Human gastric cancer (AGS), human differentiation of advanced gastric cancer (MGC803), human colorectal cancer (HCT116), human lung cancer (A549), human hepatocellular carcinoma (Bel7402) and human cervical cancer (HeLa) cell lines were obtained from the State Key Laboratory of Natural Resources and Functional Molecules of the Changbai Mountain (Yanbian University) and maintained in Dulbecco’s modified Eagle’s medium and RPMI Media 1640 (RPMI1640), supplemented with 10% foetal bovine serum 100 IU/ml penicillin, 100 mg/ml streptomycin and 2 mmol/l L-glutamine (Sigma) at 37 °7 in a humidified atmosphere containing 5% CO_2_.

### Determination of anticancer activity *in vitro*

All six human cancer cell lines and normal L02 cells were seeded in 96-well plates at the appropriate densities to ensure exponential growth throughout the experimental period (9 × 10^3^ cells per well) and then allowed to adhere for 24 h. The cells were then treated for 48 h with four serial dilutions (100, 50, 10, and 1 µM) of each compound. 5-Fluorouracil (5-FU) was used as the positive control. After incubation for 48 h, 10 µl MTT solution was added to each well to give a final concentration of 2 mg ml^−1^. The plates were then incubated for further 4 h. After incubation, the MTT solution was removed and 150 µl DMSO was added to each well to solubilise the formed product. To ensure complete solubilisation, the plates were shaken vigorously for 10 min at room temperature. The optometric density (OD) was read on a microplate reader (ELx800, BioTek, Highland Park, Winooski, VT) at a wavelength of 492 nm and the data were subsequently analysed. The percentage of cell growth inhibition was calculated from the following equation: inhibitory rate (%) = [1–(OD_treated_–OD_blank_)/(OD_control_–OD_blank_)] × 100.

### Analysis of the cell cycle distribution and apoptosis by flow cytometry

HCT116 cells were plated in six-well plates (1.0 × 10^6^ cells per well) and incubated at 37 °7 for 12 h. Exponentially growing cells were then incubated with compound **2p** at different concentrations (0 , 5.0 µM) and oridonin at 5.0 µM. After 48 h, the cells were centrifuged at 1000 rpm for 10 min and then fixed in 70% ethanol at −20 °0 for at least 24 h. The cells were subsequently resuspended in phosphate-buffered saline (PBS) containing 50 µg ml^−1^ RNase A and 50 µg ml^−1^ PI. The cellular DNA content for the cell cycle distribution analysis was measured by flow cytometry using a FACSCalibur flow cytometer with Cell Quest software (Becton-Dickinson, Franklin Lakes, NJ), plotting at least 30,000 events per sample. The percentage of cells in the G1, S and G2 phases of the cell cycle were determined using the ModFit LT V4.0 software package (Verity Software, Topsham, ME).

Apoptosis was detected using an Apoptosis Detection Kit (Invitrogen, Eugene, OR). Briefly, cells were plated in six-well plates (1.0 × 10^6^ cells per well) and incubated at 37 °C for 12 h. Exponentially growing cells were then incubated with compound **2p** at different concentrations (0 , 5.0 µM) and oridonin at 5.0 µM. Following 48 h of incubation, the cells were collected and washed twice with PBS, once with 1 × binding buffer, and then stained with 5 µM annexin V-FITC and 2.5 µM PI in 1 × binding buffer for 30 min at room temperature in the dark. Apoptotic cells were quantified using a FACSCalibur flow cytometer with the Cell Quest software (Becton-Dickinson).

## Results and discussion

### Chemistry

**Scheme 1.**
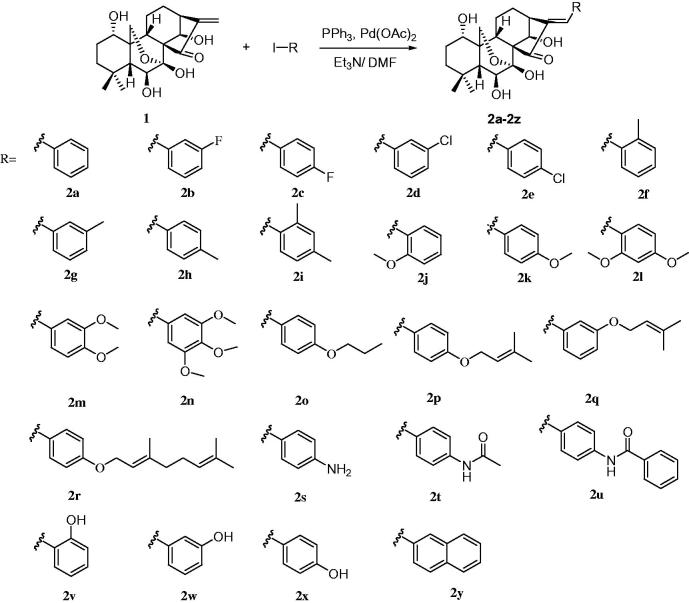
Heck reaction of oridonin with aryl iodides.

As shown in Scheme 1, oridonin (**1**) was coupled with different aryl halides under standard Heck reaction conditions [5 mol% Pd(OAc)_2_, 10 mol% PPh_3_, NEt_3_, DMF, 90 °0]. The reaction proceeded smoothly at the *α*,*β*-unsaturated ketone system of oridonin (**1**) to specifically yield E-olefin products (**2a**–**2y**)^22^. Before biological evaluation, the compounds were characterised via IR, ^1^H-NMR, and ^13^C-NMR spectrometry as well as high-resolution mass spectrometry.

### Biological evaluation

#### MTT assay and structure-activity relationship (SAR) studies

Antiproliferative activities of the synthesised compounds were evaluated against six human cancer cell lines (AGS, MGC803, Bel7402, HCT116, A549, and HeLa) and compared with those of oridonin and 5-FU. The MTT assay was performed using a standard protocol^23^.

The results of this screening are summarised in Table 1. Most of the tested compounds exhibited similar or better bioactivities *in vitro* than 5-FU. Among them, five alkoxyphenyl-substituted compounds (**2j**, **2k**, **2l**, **2p**, and **2q**) and two methylphenyl substituted compounds (**2h** and **2i**) displayed lower IC_50_ values (2.87–5.26 µM) than oridonin (8.35 µM) in human gastric cancer cells (AGS). The order of antitumour activity was *o*-OCH_3_ > *p*-OCH_3_ > *m*-isopentenyloxy > *p*-isopentenyloxy > *2,4*–CH_3_ > *p*–CH_3._ Analogously, chlorophenyl-substituted compounds (**2d** and **2e**) and alkoxyphenyl-substituted compounds (**2k**, **2l**, **2p**, **2q**, and **2r**) exhibited better antiproliferative activities (IC_50_ = 2.13, 7.27, 2.79, 3.49, 4.54, 9.10, and 6.86 µM, respectively) against human advanced gastric cancer cells (MGC803) than oridonin (IC_50_ = 9.18 µM). Among the tested compounds, the order of antitumour activities against hepatocellular carcinoma cells (Bel7402) was *2,4*-OCH_3_ > *3,4*-OCH_3_ > *p*-isopentenyloxy > *o*-OCH_3_ > *p*-OCH_3_ > *p*-propoxy > *p*-NH_2_ > *p*-phenylacetamide > *p*-geranyloxy > *2,4*–CH_3_ = *m*-isopentenyloxy; these compounds exhibited IC_50_ values of 2.41–8.62 µM, whereas the IC_50_ value of oridonin was 9.59 µM. Analysis using human colorectal cancer cells (HCT116) revealed that compound **2p** showed the highest activity (IC_50_ = 1.08 µM), followed by compounds **2l**, **2r**, **2k**, **2o**, **2u**, and **2j** with IC_50_ values of 2.28, 2.39, 2.99, 3.09, 4.02, and 5.08 µM, respectively. Therefore, the order of antiproliferative activities was *p*-isopentenyloxy > *2,4*-OCH_3_ > *p*-geranyloxy > *p*-OCH_3_ > *p*-propoxy > *p*-phenylacetamide > *o*-OCH_3._ Overall, 14 compounds (**2d**, **2e**, **2h**, **2j**–**2r**, **2u**, and **2y**) showed more potent antiproliferative activities than oridonin (IC_50_ = 24.66 µM) in human lung cancer cells (A549); compound **2r** was the most potent with an IC_50_ value of 4.16 µM. Compounds **2i**, **2n**–**2r**, and **2u** against human cervical cancer cells (HeLa) exhibited better antiproliferative activities (IC_50_ = 14.33, 8.89, 8.21, 6.33, 5.43, and 9.85 µM, respectively) than oridonin (IC_50_ = 15.03 µM). Overall, compounds **2l** (*2,4*-OCH_3_) and **2p** (*p*-isopentenyloxy) showed more potent antiproliferative activities *in vitro* than oridonin against all the six cancer cell lines. Remarkably, compound **2p** was 6.8-fold more active than oridonin, inhibiting HCT116 cell proliferation with an IC_50_ value of 1.05 µM. However, three phenol-substituted compounds (**2v**, **2w**, and **2x**) and two aminophenyl-substituted compounds (**2s** and **2t**) displayed poor antiproliferative activities against the six tumour cell lines.

**Table 1.** Antiproliferative efficacy of oridonin derivatives of **2a–2y** in six human cancer cell lines^b^.

	IC_50_ values (μM)^a^					
Compound	AGS	MGC803	Bel7402	HCT116	A549	Hela
**2a**	>100	9.13 ± 0.82	4.97 ± 0.56	47.12 ± 4.32	>100	>100
**2b**	13.86 ± 1.50	23.63 ± 1.48	>100	36.55 ± 2.43	47.43 ± 3.49	>100
**2c**	14.35 ± 2.43	>100	12.60 ± 1.05	10.25 ± 1.12	59.58 ± 4.75	>100
**2d**	14.13 ± 1.65	2.13 ± 0.13	16.94 ± 1.30	19.78 ± 3.19	12.73 ± 1.56	15.19 ± 1.47
**2e**	15.35 ± 1.78	7.27 ± 0.74	9.66 ± 1.15	13.95 ± 1.94	23.08 ± 3.63	>100
**2f**	>100	22.77 ± 1.25	>100	59.03 ± 3.37	>100	>100
**2g**	10.83 ± 2.39	17.65 ± 1.13	24.22 ± 2.27	14.16 ± 1.31	66.89 ± 5.29	22.32 ± 1.92
**2h**	5.26 ± 1.09	27.20 ± 2.38	11.29 ± 0.98	10.10 ± 1.39	18.04 ± 1.49	28.74 ± 2.13
**2i**	4.79 ± 0.94	11.51 ± 1.05	8.62 ± 0.78	8.03 ± 1.09	>100	14.33 ± 1.16
**2j**	2.87 ± 0.58	10.4 ± 1.29	3.56 ± 0.52	5.48 ± 0.64	7.89 ± 0.99	24.72 ± 2.08
**2k**	2.91 ± 0.42	2.79 ± 0.38	4.52 ± 0.46	2.99 ± 0.32	16.79 ± 2.51	68.81 ± 5.32
**2l**	5.85 ± 1.55	3.49 ± 0.44	2.41 ± 0.37	2.28 ± 0.54	5.12 ± 0.49	14.29 ± 2.04
**2m**	17.64 ± 2.32	17.68 ± 1.67	3.15 ± 0.64	11.07 ± 1.83	13.60 ± 1.89	30.66 ± 2.90
**2n**	17.30 ± 2.41	27.96 ± 1.73	14.56 ± 1.21	18.64 ± 1.78	15.78 ± 1.47	8.89 ± 0.94
**2o**	10.01 ± 1.88	10.86 ± 1.39	6.54 ± 0.94	3.09 ± 0.41	15.29 ± 1.31	8.21 ± 1.20
**2p**	4.09 ± 0.65	4.54 ± 0.94	3.21 ± 0.58	1.05 ± 0.23	5.90 ± 0.52	6.33 ± 0.96
**2q**	3.10 ± 0.41	9.10 ± 1.72	8.62 ± 1.12	7.63 ± 0.81	4.66 ± 0.30	5.43 ± 0.85
**2r**	13.7 ± 2.63	6.86 ± 1.24	8.45 ± 1.32	2.39 ± 0.19	4.16 ± 0.45	9.85 ± 2.03
**2s**	51.03 ± 3.34	53.22 ± 4.05	7.00 ± 1.05	10.04 ± 1.13	99.8 ± 7.45	>100
**2t**	22.05 ± 1.04	10.04 ± 0.84	14.56 ± 1.66	47.9 ± 3.91	97.1 ± 5.93	>100
**2u**	12.03 ± 1.99	12.22 ± 0.94	7.35 ± 1.28	4.02 ± 0.33	21.48 ± 1.88	9.79 ± 1.08
**2v**	30.28 ± 2.24	32.86 ± 2.31	60.32 ± 4.14	28.17 ± 2.67	>100	>100
**2w**	>100	>100	>100	22.14 ± 3.28	>100	>100
**2x**	>100	>100	>100	30.12 ± 3.57	>100	>100
**2y**	55.04 ± 4.43	20.96 ± 1.24	51.21 ± 5.43	17.46 ± 1.44	12.6 ± 1.39	26.02 ± 1.48
**Oridonin**	8.35 ± 0.41	9.18 ± 1.01	9.59 ± 1.19	6.84 ± 0.98	24.66 ± 2.11	15.03 ± 2.01
**5-FU**	29.61 ± 3.40	30.52 ± 4.42	21.3 ± 2.43	24.80 ± 2.08	23.65 ± 3.14	34.61 ± 3.14

a IC_50_: concentration that inhibits 50% of cell growth.

b MTT methods; cells were incubated with indicated compounds for 48 h (means ± SD, *n* = 3).

Based on these preliminary results, we identified the following structure-activity relationships: (1) the introduction of benzene with hydrophobic groups, such as alkoxy, methyl, and halogen, at the C17 position of oridonin, significantly improves antitumour activity with the order of positive potency as alkoxy > methyl > halogen; and (2) the introduction of benzene with hydrophilic groups, such as –OH and –NH_2_, reduces the antitumour activity of oridonin. Based on this interpretation, we suspected that the lipid partition coefficient crucially affects the antitumour activity of oridonin derivatives.

#### Selective inhibition of cancer cell growth by compounds 2l and 2q

Lack of selective cytotoxicity is the main factor that restricts the dose of most conventional chemotherapeutic agents^24^. We compared the toxicity of compounds **2l** and **2p** with oridonin or 5-FU on human normal liver cells (L-02). Selectivity indexes between cancer cells and L-02 cells were calculated. As shown in Table 2, compound **2p** exhibited 6.5-fold higher selectivity for HCT116 cells than for normal L-02 cells; this selectivity displayed by **2p** was significantly higher than that displayed by oridonin. Therefore, compound **2p** was further analysed to identify its mechanism of selective cytotoxicity.

**Table 2.** *In vitro* antiproliferative activities of compounds **2l** and **2p** against normal cell line (L02).

	(IC_50_, μM)^a^	Selectivity index^b^					
Compound	L02	AGS	MGC803	Bel7402	HCT116	A549	Hela
**2l**	7.15 ± 1.36	1.22	2.05	2.97	3.14	1.40	0.44
**2p**	6.88 ± 1.12	3.28	0.75	0.72	6.55	1.17	1.09
**Oridonin**	6.97 ± 0.98	0.83	0.76	0.73	1.02	0.28	0.46
**5-FU**	19.12 ± 1.01	0.65	0.63	0.90	0.65	0.81	0.55

a IC_50_: concentration that inhibits 50% of cell growth.

b SI: selective index (IC_50_ on normal cells/IC_50_ on tumour cells).

#### Cell cycle regulation by compound 2p

**Figure 2.**
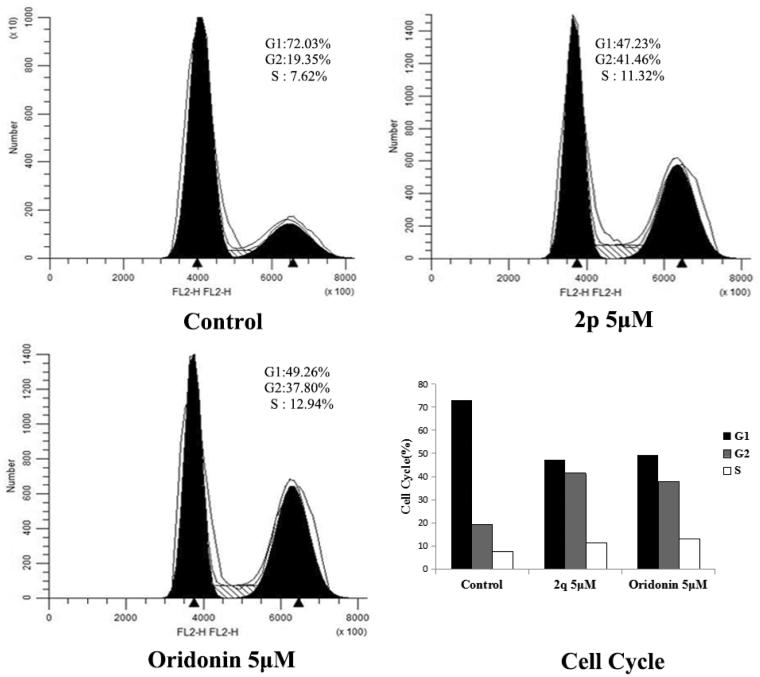
Flow cytometry analyses of cell cycle distribution of HCT116 cancer cell after treatment of compound **2p** (5.00 μM), oridonin (5.00 μM) and no treatment (Ctrl) as a reference control for 48 h.

Numerous cytotoxic compounds exert their antiproliferative effect by inducing cell cycle arrest (at a particular cell cycle checkpoint), apoptosis, or both^25^. These mechanisms are considered to be effective anticancer strategies^26^. We used fluorescence-activated cell sorting analysis to explore the mechanism by which compound **2p** reduced the viability of HCT116 cells. Compound **2p** and oridonin were selected and tested against HCT116 cell lines at a concentration of 5 µM. As shown in Figure 2, compound **2p** significantly increased the percentage of G2 cell population from 19.35 to 41.46% after 48 h of incubation, whereas oridonin increased it to 37.80%. This finding suggests that compound **2p** induces cell cycle arrest at the G2 phase.

#### Apoptotic effects of compound 2p

**Figure 3.**
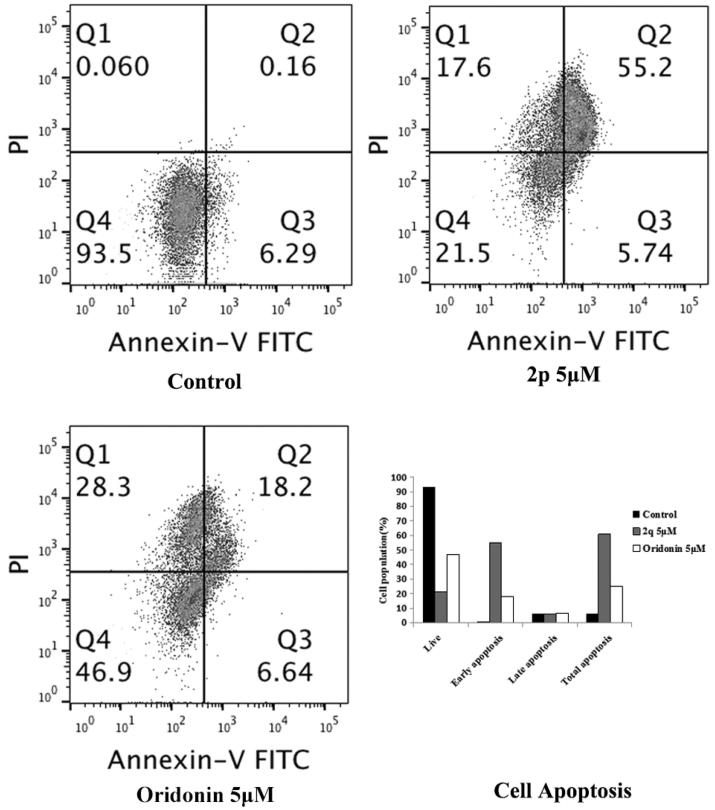
Apoptosis induction in HCT116 cancer cell after treatment with **2p** (5.00 μM), oridonin (5.00 μM) and no treatment (Ctrl) as a reference control for 48 h.

Because compound **2p** arrested the cells in the G2 phase, we investigated whether it also causes apoptosis. HCT116 cells were treated with the vehicle, compound **2p** (5 µM), or oridonin (5 µM) for 48 h, and then stained with annexin V-FITC and PI. As shown in Figure 3, the percentage of total apoptotic cells (right quadrants, Q_2_ + Q_3_) increased to 60.94% after treatment with compound **2p**, whereas it was 24.84% after oridonin treatment; vehicle treatment induced apoptosis in 6.20% of the cells. This result indicated that compound **2p** is a more potent inducer of apoptosis than oridonin.

## Conclusion

A series of oridonin derivatives with substituted benzene analogues at the C17 position were designed, synthesised, and evaluated for their antiproliferative properties against six human cancer cell lines (AGS, MGC803, Bel7402, HCT116, A549, and HeLa), and the noncancerous human L02 cells. Most of the synthesised oridonin derivatives displayed significant antiproliferative effects in these cancer cell lines. SAR analysis indicated that the alkoxyphenyl ring at the C17 position of oridonin effectively improves its antitumour efficacy. Compound **2p** possessed the highest antiproliferative activity against HCT116 cells; it was 6.8-fold more potent than oridonin. The IC_50_ value of **2p** in L02 cells was 6.5-fold higher than that in HCT116 cells, indicating that it exhibits selective antiproliferative effects.

In addition, cell cycle analysis revealed that compound **2p** arrested HCT116 cells at the G2 phase. It increased the percentage of apoptotic cells to a greater extent than oridonin. Therefore, compound **2p** could serve as a promising lead candidate for further studies.
